# Adenosine A_2A_ Receptor Antagonists in Neurodegenerative Diseases: Huge Potential and Huge Challenges

**DOI:** 10.3389/fpsyt.2018.00068

**Published:** 2018-03-12

**Authors:** Rafael Franco, Gemma Navarro

**Affiliations:** ^1^Department of Biochemistry and Molecular Biomedicine, School of Biology, Universidad de Barcelona, Barcelona, Spain; ^2^Centro de Investigación en Red, Enfermedades Neurodegenerativas (CIBERNED), Instituto de Salud Carlos III, Madrid, Spain; ^3^Department of Biochemistry and Physiology, Faculty of Pharmacy, Universitat de Barcelona, Barcelona, Spain

**Keywords:** neuroprotection, astroglia and microglia, neuronal death, Alzheimer’s and Parkinson’s disease, human, clinical trials as topic, caffeine

## Background

In this opinion paper, we provide scientific-based reasons about the huge therapeutic potential of adenosine A_2A_ receptor antagonists, and about the huge challenges to demonstrate efficacy in clinical trials, i.e., to provide data now required to approve a new medication by the regulatory bodies, such as U.S. Food and Drug Administration (FDA).

Adenosine is an autacoid present in all tissue and body fluids. Adenosine, whose extracellular concentration is controlled by producing/degrading enzymes and by nucleoside transporters, acts *via* four (A_1_, A_2A_, A_2B_, and A_3_) specific cell surface receptors that belong to the superfamily of G-protein-coupled receptors. For decades, adenosine receptors have shown promise as targets of medications for a variety of ailments. Until recently, however, the only approved medicine was adenosine itself, i.e., the endogenous agonist, to combat arrhythmias, such as paroxysmal supraventricular tachycardia (1–3). Prospects are changing as the first medication targeting selectively the adenosine A_2A_ receptor has been approved few years ago in Japan. The recently approved drug is an antagonist, i.e., a receptor blocker (see later). A_2A_ receptor antagonists show promise in neuroprotection, although for Huntington’s or Niemann Pick’s diseases it is suggested that antagonists may be detrimental and/or there is controversy on which is the efficacious intervention, i.e., receptor activation or blockade [see Ref. (4–9) and references therein].

## Potential of A_2A_ Receptor Ligands in the Therapy of Neurodegenerative Diseases

At present, not only the A_2A_ receptor (A_2A_R) is at the center stage for increasing the therapeutic tools in a variety of clinical indications, but this opinion paper focuses on the A_2A_R antagonists, which shows promise in immune-mediated control of cancer progression (10–13), in atrial fibrillation (14, 15), and in fighting against neurodegenerative diseases (see later). It is relevant that virtually all the selective A_2A_R antagonists whose toxicity has been tested in animal models are very safe. Safety has been confirmed in the clinical trials performed using different structures [e.g., Ref. (16, 17)]. Istradefylline (KW-6002) is one of the most studied antagonists; it is safe and efficacious in Parkinson’s disease. Accordingly, it was approved in Japan in 2013 for adjunctive treatment of Parkinson’s patients (under the *Nouriast*™) (18–20). To our knowledge, up to five clinical trials with different antagonists were or are being undertaken (18, 21), but none of them has yet got the approval by the U.S. FDA. In our opinion, the two main reasons of the difficulties in translating very promising preclinical assays into medications are (i) the tight requirements and (ii) the urgent need of efficacious approaches to assess neurodegeneration/neuroprotection in humans. It is commented in drug discovery forums that quite a number of current drugs could not pass today the tight requirements posed by the regulatory bodies. The issue of assessing how to measure the neuroprotective efficacy of a drug is commented later.

Parkinson’s and Alzheimer’s are the most extended neurodegenerative diseases in modern societies with high life expectancy (22, 23). With some exceptions of early-onset symptoms (24), age is the main factor for triggering the clinical symptoms (25–28). Whereas, Parkinson’s disease patients have successful dopamine-replacement therapies and other tools that may be used in the disease progression or to decrease the appearance of the medication side effects (29–31), Alzheimer’s disease patients have not yet got any really efficacious therapeutic drug/tool (32, 33).

Clinical manifestation of Parkinson’s disease occurs when a significant number of nigral dopamine-producing neurons have disappeared. Natural aging leads to 18% of loss of tyrosine hydroxylase-positive neurons in the nigra, whereas the degree of denervation in patients is very wide, going from 50 to 90%, even shortly after diagnosis (34). In this study in post-mortem samples, the authors state: “*with several of the short-duration subjects showing comparable, severe loss of tyrosine hydroxylase-positive neurons to that seen in subjects 20 years, post-diagnosis*” (34). The idea behind the use of A_2A_R antagonists in this disease is the adenosine-dopamine antagonism (35–37) in the striatum, where the expression of A_2A_Rs is highest in the whole mammalian body (38). Therefore, dopamine-replacement therapy may be potentiated by the blockade of the A_2A_R. Indeed, Nouriast™ may serve to achieve efficacy of dopamine-replacement therapies at lower levels of dopaminergic drugs, such as levodopa. But the key point is that whereas levodopa is not neuroprotective, several preclinical assays indicate that A_2A_R antagonists show neuroprotective effects [see Ref. (39–42)]. Moreover, transgenic A_2A_R animals are more resistant to neurodegeneration induced by either 1-methyl-4-phenyl-1,2,3,6 tetra-hydropyridine (MPTP)-induced nigral lesion or focal cerebral ischemia (43–46). Further to those preclinical results, epidemiological studies showed that caffeine, which is a mixed/non-selective adenosine receptor antagonist, decrease the risk of suffering from Parkinson’s or from Alzheimer’s diseases [(47, 48); see Ref. (49) and references therein for review]. In summary, one challenge for the progression of A_2A_R antagonist into approved drugs is to demonstrate neuroprotection in humans, for instance to decrease the death rate of nigral dopaminergic neurons.

## Challenges in Developing Neuroprotective Medicines

Based on development of our profession of translational research we notice that, often, scientific reports do not distinguish among symptom improvement and disease modifying drugs. Any drug that “improves” the condition of a parkinsonian animal model may be considered as neuroprotective. When the drug faces a clinical trial with patients, the promoter has to decide between analyzing symptom improvement and disease progression as primary outcome measure. For reasons further specified below, clinical trials for common neurodegenerative diseases analyze symptoms as primary outcome with a further difficulty: for ethical reasons patients should continue to take the *ad hoc* medication. Therefore, it is difficult to address whether any new medicine is improving symptoms or decreasing the side effects. For instance, parkinsonian patients recruited for clinical trials continue taking levodopa; thus, the assayed new drug must improve symptoms or reduce side effects appearing after years of taking levodopa. Difficulties in approving disease modifying, i.e., neuroprotective, medication for Parkinson’s were already noticed and reviewed years ago (50). Although the issue is on the table, to our knowledge there is not any effective action for neurodegenerative diseases unlike the case of rare diseases for which drug approval may be accelerated. There is an apparent lack of consensual rules to evaluate, and consequently approve, drugs whose action is to prevent neuronal death, i.e., drugs that modify the progression of a neurodegenerative disease. Apart from hoping for specific measures tackling this issue at the regulatory level, we think that (i) we are quickly moving to have reliable neuroprotection biomarkers with positron emission tomography resolution, i.e., to monitor neurodegeneration *in vivo* in humans and (ii) regulatory bodies should consider the approval of the use of safe drugs in healthy cohorts to assess the long-term potential as neuroprotectants (assumption would be that drug should delay clinical symptoms and/or delay neurodegenerative disease progression). The latter may seem risky but, again in our opinion, not riskier than taking food supplements or drug supplements to “combat aging,” such as testosterone.

## Challenges in Designing Neuroprotective A_2A_R Antagonists

The two sides of drug action, i.e., addressing symptoms and disease progression, should be considered for any drug type (A_2A_R antagonists in this article). Whereas in the case of Parkinson’s disease, the targets of antagonists in adjunctive treatments of dopamine-replacement therapies are A_2A_R receptors in neurons, the A_2A_ receptor containing targeted cells in the MPTP model of Parkinson’s disease that could be responsible for the neuroprotective action could not be determined in transgenic animals with cell-type-specific (conditional) deletion of the receptor (46). In this scenario, and based in convergent and wide experimental evidence, we consider that the role of the A_2A_ receptor expressed in microglia should not be neglected. Hence, another challenge is to select (for a given disease) the receptor to be targeted, but also the cell where the receptor must be targeted and/or when the receptor of a given cell (neuron or glia) must be targeted to afford neuroprotection. Years ago, we found a relevant A_2A_R-related side result on studying gene expression in samples from Alzheimer’s patients. Genomics-relevant results were upregulation in samples from patients of the Kv3.4 voltage-gated potassium channel (51) and of the adenosine A_1_ receptor (52). Interestingly, when we moved to perform immunostaining in the cerebral cortex and hippocampus from necropsies of Alzheimer’s disease (AD) patients, we confirmed the upregulation of the adenosine A_2A_ receptor concomitant with a change in the pattern of expression. The receptor was found *inter alia* in degenerating structures, i.e., in both dystrophic neurites and neurons exhibiting neurofibrillary tangles. Whereas, specific mRNA expression in the assayed brain areas was not different from that found in control samples, the microglial expression of the protein was negligible or absent in control samples but was evidently expressed in microglial cells in both the cerebral cortex and the hippocampus of patients (52).

Function of immune cells in the periphery and of microglia in the CNS is regulated by the A_2A_R. Due to the extensive work of M. Sitkovsky’s and other laboratories devoted to targeting adenosine receptors to combat cancer, the already promising immunotherapy to combat certain tumor types, may be enhanced by the blockade of A_2A_R expressed in immune cells (10–13). Provided that ATP is degraded to adenosine in oxygen partial deficiency and/or cell death occurring in degenerating environments, increased adenosine levels activate upregulated microglial A_2A_Rs. Therefore, an obligate drug discovery approach is to target those cells and those receptors that promote M2-skewed microglial responses.

We highlight microglia as the most likely cell type to be targeted (for neuroprotection) by A_2A_R antagonists. Straightforward data in different models, support the view that A_2A_R activation in glia drives neuroinflammation and, therefore, the selective blockade of this receptor may be neuroprotective (40, 53–58). We think that more experimental effort is required to define when and how A_2A_R antagonists may achieve conversion from M1-skewed (proinflammatory) to M2-skewed (neuroprotective) microglia, something that requires control of the production of cytokines/chemokines, interferon-gamma, etc. [see (59) for review]. Intrinsic to any transformation, in this case into M1 or into M2 cells, there is a time window of opportunity whose starting point and duration should be also explored. In other words, when to start the application of the intervention and when it is too late.

We would like to end this paper with a further opinion, which A_2A_R antagonists may “conceptually” be the new beta-blockers (ß-adrenergic antagonists) whose therapeutic potential is vast from cardiovascular problems to asthma.

## Author Contributions

The two authors participated in the conceptual design of this opinion article. RF did literature search. The two authors contributed to the writing and carefully checked English spelling/grammar.

## Conflict of Interest Statement

The authors declare that the research was conducted in the absence of any commercial or financial relationships that could be construed as a potential conflict of interest.
